# To assess the knowledge, attitude and practices in biomedical waste management among health care workers in dedicated COVID hospital in Bangalore

**DOI:** 10.1186/s43162-021-00066-9

**Published:** 2021-11-16

**Authors:** T. J. Basavaraj, B. L. Shashibhushan, Amala Sreedevi

**Affiliations:** Department of Pulmonary Medicine, Victoria Hospital, Bangalore Medical College and Research Institute, Bangalore, Karnataka India

**Keywords:** Biomedical waste management, COVID-19, Health care workers

## Abstract

**Background:**

In this pandemic of COVID-19, the highest amount of infective material, biomedical waste is generated in hospitals and it is frequently handled by the healthcare workers irrespective of cadres. Hence the awareness of healthcare workers in regards with biomedical waste (BMW) management is crucial in this pandemic. This study is therefore conducted to assess the knowledge, attitude and practices in BMW management among health care workers in our institution.

**Results:**

A total of 280 subjects consisting of doctors, nursing staff and group D workers were included in the study after obtaining informed consent. The knowledge among healthcare workers was satisfactory, but comparatively group D workers were lagging behind. Overall they all have a good attitude towards BMW management but practices on BMW management needs improvement mostly among group D workers.

**Conclusions:**

There have to be regular training programmes on biomedical waste management and its hazards for all the healthcare workers including group D workers. Along with educational intervention, strict implementation of biomedical waste management guidelines with its monitoring at all levels is also very much essential.

## Background

The world is now being crumpled in the hands of the novel coronavirus (SARS-CoV-2) by the coronavirus disease 2019 (COVID-19). It emerged in Wuhan, Hubei Province, China, as a cluster of pneumonia on 31 December 2019 as reported by the World Health Organization (WHO) and eventually was declared as a pandemic on 11 March 2020 by the WHO [1]. The first case of COVID-19 in India was detected on the 31st of January in Kerala state and since then, the numbers have been ever increasing.

COVID-19, being a highly contagious disease, can only be prevented by proper hygiene and practices like social distancing. Especially in designated COVID-19 hospitals, which will be producing the highest amount of infective material, the biomedical waste has to be adequately managed along with proper monitoring. Biomedical waste (BMW) means any waste, which is generated during the diagnosis, treatment or immunisation of human beings or animals or research activities pertaining thereto or in the production or testing of biological or in health camps [2]. Its management begins from the initial stage of generation of waste, segregation at the source, storage at the site and disinfection, and transfer to the terminal disposal site plays a critical role in the disposal of waste. Health care workers are the first in line in managing the BMW [3]. Therefore, their knowledge, attitude and practices (KAP) regarding BMW plays a crucial role in its proper management and its mismanagement not only affects the hospital but also the general population by and large.

Therefore, this study is conducted to assess the knowledge, attitude and practices in BMW management in a designated COVID-19 hospital in Bangalore so as to rectify the lacunae that have been identified from this study for the better management of the hospital.

## Methods

The study is a questionnaire-based observational study conducted on the health care workers attending the COVID-19 screening OPD post duty in the COVID-19 wards and COVID-19 screening OPD in Victoria Hospital, Bangalore Medical College and Research Institute (BMCRI), Bangalore, during their 14-day quarantine during May–July 2020. The study included 280 subjects comprising 112 doctors, 107 nursing staff and 61 group D workers after obtaining informed written consent. This study was approved by the ethics committee of Bangalore Medical College and Research Institute with approval number BMCRI/PS/092020-21.

## Result

In this questionnaire-based observational study, we found out that doctors and nurses have better knowledge regarding biomedical waste management than group D workers. But both doctors and group D did not have adequate training in this regard. Most of them considered biomedical waste as hazardous and knew segregation based on colour coding is the most important part in BMW management and that PPE reduces the risk of infection. Table 1 shows the knowledge of health care workers on BMW management.

**Table 1:** Knowledge of health care workers on BMW management

SL NO	Knowledge on BMW management	Doctors (*N* = 112) *N*%	Nurses (*N* = 107) *N*%	Group D (*N*= 61) *N*%
1	Have you undergone any training in BMW management?	36.6	100	14.75
2	Is there any hazard associated with BMW management	100	58.8	26.22
3	Do you know the symbol for biohazard	100	100	100
4	Most important aspect of BMW management is segregation	100	100	77.04
5	PEP can be taken at anytime	0	0	0
6	Do you know about the colour coding system for segregation	100	100	100
7	General wastes are to be collected in yellow bin	0	0	100
8	Wearing PPE reduces risk of infection	100	91.58	100
9	Maximum storage time for untreated waste is 2 days or 48 h	91.07	16.82	77.04
10	Yellow bag is treated by incineration	86.60	100	45.23

In general, all health care workers had a good attitude towards BMW management, and the results are shown below in Table 2.

**Table 2:** Attitude of health care workers on BMW management

SL NO	Attitude on BMW management	Doctors (*N* = 112) *N*%	Nurses (*N* = 107) *N*%	Group D (*N* = 61) *N*%
1	Proper BMW management is an issue	73.21	25.23	60.65
2	Safe BMW management need team work	100	100	100
3	General public health can be adversely affected by BMW	100	75.70	67.21
4	Is needle stick injury/sharp injury a concern	100	100	80.32
5	BMW should be segregated at the point of origin	100	100	100
6	Do you think BMW management and handling should be a compulsory part of the curriculum	100	100	100
7	Proper BMW disposal can prevent infection transmission	100	100	100
8	Reporting of needle stick injury is an extra burden on work	0	25.23	100
9	Colour code bag use for waste segregation is a must	100	100	100
10	For persons involved in BMW handling occupational safety is a must	100	100	100

We found out in our study that PPE was not worn properly by any of the group D who participated in the study, which is of concern. Segregation by colour coding was not followed by most of the group D and most of them do not report accidents and injuries that happen in the workplace. All the nursing staff who participated in followed post exposure prophylaxis (PEP) after needle stick injury unlike the doctors and group D workers. Records on handling BMW were better maintained by nursing staff and group D workers than doctors. Most of the healthcare workers were immunised for hepatitis B infection. Table 3 shows the results acquired from the study on practices of healthcare workers on BMW management.

**Table 3:** Practices of health care workers on BMW management

SL NO	Practices on biomedical waste management	Doctors (*N* = ) *N*%	Nurses (*N* = ) *N*%	Group D (*N* = ) *N*%
1	Do you wear PPE while handling BMW	91.96	100	0
2	Do you segregate BMW at the point of into different categories	100	100	100
3	Do you use puncture-proof plastic containers to collect waste sharps	100	100	96.72
4	Do you follow colour coding for segregation of waste	100	100	0
5	Do you maintain a record for BMW at the point of origin	73.21	87.5	95.08
6	Do you have a system of reporting injuries and accidents	95.53	100	50.81
7	Have you been immunised against Hepatitis B	100	100	70.49
8	Do you follow PEP after needle stick injury or percutaneous injury	91.07	100	40.98
9	Do you put non-infectious wastes in black container	100	87.5	100
10	Do you know the method to prepare 1 L off 1% Sodium hypochlorite from available 5% strength	86.60	100	60.65

## Discussion

This study was conducted to assess the knowledge, attitude and practice of BMW management among healthcare workers in our hospital which is a COVID-19 dedicated hospital in Bangalore. The knowledge regarding biomedical waste management among healthcare workers who participated in this was found to be satisfactory. However, the knowledge was better among doctors and nurses when compared to group D workers. The findings were similar as observed in various other studies [4–6].

All the participants in our study were well aware of colour coding for waste segregation which is in contrast to the observations by Mathur V et al. where sanitary workers knowledge of colour coding was poor [6]. Another study by Soyam GC et al. also observed Group D having poor knowledge of colour coding when compared to nursing staff which is in contrast to our study [3]. All the participants in our study including group D workers could identify the symbol of biohazard whereas in the study by Anand et al only 52.7% nurses and 20% of the class IV workers could identify the symbol of biohazard [4]. Another study by Malini et al observed identification of biohazard symbol by 61.7% nurses only which is in contrast to our study [5]. Mehta et al did observe that 42.46% doctors and only 15.9% nurses knew about when to take post exposure prophylaxis (PEP) [7]. In our study, all the participants did agree that PEP cannot be taken at anytime.

Majority of the participants in our study largely had a positive attitude towards biomedical waste management. All the participants felt biomedical waste management as an issue and necessity to work in a team for safe disposal and management of biomedical waste. Majority of the participants shared the same opinion in a study by Malini et al. [5]. Similarly, in another study by Anand et al, most of the doctors and nursing staff considered biomedical waste management as team work which is similar to our study [4]. In another study by Sharma et al. it was observed that educational intervention resulted in a similar observation which included nursing staff and lab technicians as study population [8].

In this study, though all the doctors and majority of nursing staff and group D workers felt that biomedical waste can adversely affect the health of the general public, some percentage of nursing staff and group D workers did not agree with it. Lavanya et al in their study observed that only 51.9% of the nursing staff and 82.1% housekeeping staff considered biomedical waste as infectious [9]. Malini et al., in their study, found that the majority of the participants felt biomedical waste to be hazardous [5].

Needle stick injury was a concern for all the doctors and nursing staff in our study. Though the majority of the group D workers had the same concern, but some of them had a lax attitude towards it. Malini et al had a similar observation in their study where the majority of doctors and nursing staff did consider needle stick injury a concern [5].

We observed more rational biomedical waste management practice among doctors and nurses when compared to group D workers. In our study, it was observed that group D workers did not wear PPE always while handling biomedical waste unlike nurses and doctors. Whereas Lavanya et al. in their study found 60.7% housekeeping staff practising personal protective measures while handling biomedical waste which was less than nursing staff [9]. In the current COVID-19 pandemic situation, it is more important to take personal care while handling biomedical waste. Fomite transmission is one of the modes of severe acute respiratory syndrome coronavirus 2 (SARS-CoV-2) infection transmissions [10]. Hence an adequate personal protective measure has to be taken while handling biomedical waste.

Practice of maintaining record for biomedical waste by doctors was less when compared to nurses and group D in our study. Similarly, Dey et al. observed that the practice of maintaining a record for biomedical waste was more with nursing staff when compared to resident doctors [11].

Doctors and nurses in our study are aware of reporting injuries and accidents whereas only about 50% of the group D workers were aware about this. The practice of reporting injuries due to sharps was seen in less than 50% of the participants in a study by Anand et al. that included doctors, nurses, lab technicians and class IV employees [4]. Also, Lavanya et al observed the lowest percentage of reporting of accidents among housekeeping staff [9]. Practice of the following PEP following needle stick injury was lowest among group D workers in our study when compared to doctors and nurses.

Hepatitis B vaccination was received by all doctors and nurses in our study and about 29.5% group D workers were not vaccinated. Similarly, Anand et al. observed the lowest vaccination with hepatitis B among Class IV employees [4]. Imchen et al. in their study found that 70.6% nursing staff being vaccinated for hepatitis B [12] unlike in our study where all nursing staff were vaccinated for hepatitis B. Also, Malini et al. also observed that not all doctors and nurses were vaccinated for hepatitis B in their study [5].

All the nurses who participated in this study knew to prepare 1 L of 1% sodium hypochlorite from available 5% strength, whereas 13.3% of doctors and 39.3% of group D workers did not know the method of preparation. These findings are better than that observed by Mehta et al., as the percentage of doctors and nurses who knew the method of preparing 1% sodium hypochlorite were less than in our study [7].

## Conclusions

The COVID-19 pandemic has increased the amount of biomedical waste generated that also contains PPE and the ability of coronavirus to remain active on various surfaces for the variable period has made them hazardous. Also, fomite transmission is one of the modes of SARS-CoV-2) infection transmission [10, 13]. Hence, all the healthcare workers handling biomedical waste should be taking utmost care and personal protection.

Findings from our study reveal that though the participants in our study have a fair knowledge regarding biomedical waste management still there is a lot of scope in not only improving the knowledge but also in changing the attitude and inculcating more rational practices towards the same.

Majority of the group D workers in our study did not have any training on biomedical waste management. And, all the participants in our study felt the need of having biomedical waste management and handling as part of the curriculum. Thus, there has to be a regular training programmes on biomedical waste management and its hazards for all the healthcare workers including group D workers. A study by Sharma et al. has shown a significant improvement in knowledge and attitude of study participants towards biomedical waste management following educational intervention [8]. Along with educational intervention, strict implementation of biomedical waste management guidelines with its monitoring at all levels is also very much essential.

## Data Availability

All data generated or analysed during this study are included in this published article.

## Abbreviations

COVID-19: Coronavirus disease 2019
SARS-CoV-2: Severe acute respiratory syndrome coronavirus 2
BMW: Biomedical waste
WHO: World Health Orgnization
KAP: Knowledge, attitude, practices
OPD: Out-patient department
BMCRI: Bangalore Medical College and Research Institute
PPE: Personal protective equipment
PEP: Post exposure prophylaxis
